# Board of Health, Organization and Committees

**Published:** 1875-07

**Authors:** 


ORGANIZATION STATE BOARD OF HEALTH OF GEORGIA.

PRESIDENT—J. G. Thomas, M.D., Savannah.

SECRETARY—V. H. Taliaferro, M.D., Atlanta.

BOARD—J. G. Thomas, M.D., Savannah; George F. Cooper, M.D., Americus; Benjamin M. Cromwell, M.D., Albany; F. A. Stanford, M.D., Columbus; C. B. Nottingham, M.D., Macon; H. H. Carlton, M.D., Athens; G. W. Holmes, M.D., Rome; J. P. Logan, M.D., Atlanta; Henry F. Campbell, M.D., Augusta; the Comptroller General ex-officio; the Attorney General ex-officio; the State Geologist ex-officio.

STANDING COMMITTEES.

FINANCE—Logan, Carlton and Comptroller General.

STATIONERY AND PRINTING—Logan, Comptroller General and and State Geologist.

LEGISLATION—Attorney General, Campbell, Nottingham, Stanford and Holmes.

PRUDENTIAL—Logan, Comptroller General and Attorney General.

ENDEMIC, EPIDEMIC AND CONTAGIOUS DISEASES—Campbell, Nottingham and Cromwell.

HYGIENE OF SCHOOLS, PRISONS AND PUBLIC INSTITUTIONS—Nottingham, Stanford and Cooper.

GEOLOGY, TOPOGRAPHY, ETC.—State Geologist, Holmes and Cooper.

PRISONS AND SPECIAL SOURCES OF DANGER TO LIFE AND HEALTH—Stanford, Logan and Cooper.

At the recent meeting of the Board the following preamble and resolutions were adopted:

WHEREAS, This body is fully impressed with the great difficulty as well as the paramount importance and weighty responsibility of their relations to the public; AND WHEREAS, They clearly recognize the great value and assistance of thoughtful suggestions as promotive of the benevolent ends for which the Board has been inaugurated:

*Resolved*, That we cordially invite members of the medical profession and other scientific men to communicate freely with us, through the Secretary, on any and all subjects pertaining to the public health, and that we assure them and all public-spirited people, whether of scientific pretensions or not, that such useful suggestions will ever meet the most respectful and thankful consideration.

*Resolved*, That the above be furnished the public journals, as an indication of the wishes of this Board.

